# Variable content and distribution of arabinogalactan proteins in banana (*Musa* spp.) under low temperature stress

**DOI:** 10.3389/fpls.2015.00353

**Published:** 2015-05-27

**Authors:** Yonglian Yan, Tomáš Takáč, Xiaoquan Li, Houbin Chen, Yingying Wang, Enfeng Xu, Ling Xie, Zhaohua Su, Jozef Šamaj, Chunxiang Xu

**Affiliations:** ^1^Department of Pomology, College of Horticulture, South China Agricultural UniversityGuangzhou, China; ^2^Department of Cell Biology, Faculty of Science, Centre of the Region Haná for Biotechnological and Agricultural Research, Palacký UniversityOlomouc, Czech Republic; ^3^Department of Healthy Seeds, Institute of Biotechnology, Guangxi Academy of Agricultural SciencesNanning, China

**Keywords:** arabinogalactan proteins, abiotic stress, banana (*Musa* spp.), immuno-labeling, low temperature stress, plant cell wall, plant-environment interactions, spatial distribution

## Abstract

Information on the spatial distribution of arabinogalactan proteins (AGPs) in plant organs and tissues during plant reactions to low temperature (LT) is limited. In this study, the extracellular distribution of AGPs in banana leaves and roots, and their changes under LT stress were investigated in two genotypes differing in chilling tolerance, by immuno-techniques using 17 monoclonal antibodies against different AGP epitopes. Changes in total classical AGPs in banana leaves were also tested. The results showed that AGP epitopes recognized by JIM4, JIM14, JIM16, and CCRC-M32 antibodies were primarily distributed in leaf veins, while those recognized by JIM8, JIM13, JIM15, and PN16.4B4 antibodies exhibited predominant sclerenchymal localization. Epitopes recognized by LM2, LM14, and MAC207 antibodies were distributed in both epidermal and mesophyll cells. Both genotypes accumulated classical AGPs in leaves under LT treatment, and the chilling tolerant genotype contained higher classical AGPs at each temperature treatment. The abundance of JIM4 and JIM16 epitopes in the chilling-sensitive genotype decreased slightly after LT treatment, and this trend was opposite for the tolerant one. LT induced accumulation of LM2- and LM14-immunoreactive AGPs in the tolerant genotype compared to the sensitive one, especially in phloem and mesophyll cells. These epitopes thus might play important roles in banana LT tolerance. Different AGP components also showed differential distribution patterns in banana roots. In general, banana roots started to accumulate AGPs under LT treatment earlier than leaves. The levels of AGPs recognized by MAC207 and JIM13 antibodies in the control roots of the tolerant genotype were higher than in the chilling sensitive one. Furthermore, the chilling tolerant genotype showed high immuno-reactivity against JIM13 antibody. These results indicate that several AGPs are likely involved in banana tolerance to chilling injury.

## Introduction

Plant cell wall modification is a frequently studied phenomenon in plant-environment interactions (Sasidharan et al., 2011). Plants are frequently subjected to potentially harmful environmental conditions. As a result of adaptation, plants developed various strategies to face adverse conditions. Most abiotic stressors act upon the cell wall-plasma membrane interface. When exposed to biotic or abiotic stresses, both the composition and structure of plant cell walls as well as individual components such as pectin (Solecka et al., 2008; Ma et al., 2013), callose (Santi et al., 2013; Voigt, 2014), cellulose (Ellis and Turner, 2001; Blanco-Ulate et al., 2014), hemicellulose (Kubacka-Zêbalska and Kacperska, 1999; Blanco-Ulate et al., 2014), and structural proteins (Simon et al., 2005; Shetty et al., 2009; Sasidharan et al., 2011; Xie et al., 2011) are modified. The appropriate and timely modification of the cell wall is integral to the plant's strategy to survive against unfavorable conditions.

Plant cell walls are composed of approximately 10% structural proteins, including extensins, glycine-rich proteins, proline-rich proteins (PRPs), solanaceous lectins, and arabinogalactan proteins (AGPs) (Lamport, 1965; Showalter, 1993). Among these proteins, AGPs are hyperglycosylated and represent members of the hydroxyproline-rich glycoproteins (HRGPs), a superfamily of glycoproteins that are abundant in plant cell walls (Sommer-Knudsen et al., 1998; Showalter et al., 2010). AGPs are proposed to play important roles in a range of plant growth and developmental processes (Seifert and Roberts, 2007; Ellis et al., 2010; Lamport and Várnai, 2013; Knoch et al., 2014) and in plant-microbe interactions (Xie et al., 2011; Nguema-Ona et al., 2013).

Low temperature (LT) is one of the most common abiotic stresses, and it adversely affects the growth and development of plants growing in temperate climates, while significantly limiting their agricultural productivity and distribution (Takáč et al., 2003; Chinnusamy et al., 2007; Theocharis et al., 2012). Many studies have suggested that some HRGP members, such as extensins (Kozbial et al., 1998; Lucau-Danila et al., 2012), PRPs (Zhang and Schläppi, 2007; Gothandam et al., 2010) and glycine-rich proteins (Kim et al., 2010), play key roles in defense against LT stress. However, only a very limited number of reports have addressed the functions of AGPs during LT stress.

Banana (*Musa* spp.) is one of the most important fruit and food crops in the tropical and subtropical regions of the world. Because the temperature can be very low during winter in the subtropical regions (Turner and Lahav, 1983), banana production is often threatened by LT, which at the cellular level, causes membrane disintegration and lipid peroxidation, and thereafter growth retardation and even death, resulting in decreased banana production (Shmueli, 1960; Huang et al., 1982; Wang and Liang, 1994; Chen et al., 1999; Xu et al., 2000; Yang et al., 2012). Therefore, it is of both biological and agricultural importance to understand the molecular mechanism of banana chilling tolerance.

Immuno-histochemical techniques based on monoclonal antibodies represent efficient ways to study these proteins because they can precisely localize and monitor the abundance of diverse cell wall polymers *in situ* within complex tissues (Knox, 2008). These methods have been successfully employed to study the distribution and function of plant cell wall proteins during plant growth, development and pathogen-host interactions (Smallwood et al., 1994; Deepak et al., 2007; Pan et al., 2011; Xie et al., 2011; Xu et al., 2011a,b). These studies were performed on roots (Smallwood et al., 1994; Šamaj et al., 1998, 1999b), embryogenic cells or embryos (Šamaj et al., 1999a, 2008; Pan et al., 2011; Xu et al., 2011a), shoot apical meristems (Zhang et al., 2008), and flower components (Chen et al., 2008; Losada and Herrero, 2014). However, the information about the distribution of plant structural proteins in plant leaves is nearly absent.

To test possible involvement of AGPs in the tolerance of banana to chilling stress, a systematic immuno-fluorescence labeling approach by employing 17 monoclonal antibodies that recognized AGP epitopes was performed in banana leaves and roots under LT stress. Immuno-dot blotting was used to quantify AGP changes in banana leaves. In addition, the changes in total classical AGPs in banana leaves were also measured.

## Materials and methods

### Plant materials and LT treatment

Tissue-cultured banana plants (two genotypes, namely *Musa* spp. AAA cv. Baxijiao and *Musa* spp. ABB cv. Dajiao, which are LT-sensitive and tolerant, respectively) (Wang and Liang, 1994; Chen et al., 1999; Xu et al., 2000; Yang et al., 2012) were grown in pots containing mass medium (Tref, Germany) at normal growth conditions [28°C, 85% relative humidity, 12-h photoperiod with a PPFD of 250 μmol (m^−2.^s^−1^)]. After 3 months of growth, the plants were transferred to an acclimation chamber. The temperature setting was decreased by 3°C from 28 to 7°C every 3 days (28, 25, 22, 19, 16, 13, 10, and 7°C). Samples (three plants for each genotype and temperature) were collected at the end of the third day at 25 (the control plants), 16, 10, and 7°C. All analyses were performed on laminas of the youngest fully-developed leaves. The terms “leaf” and “lamina” were used synonymously in this text.

To observe the changes in AGP levels in banana roots, banana plantlets cultured in rooting medium for 3 weeks were used. Both genotypes were treated by LT as described above for leaves.

### Extraction of pectin and AGP from banana leaves and AGP measurement

Pectin was extracted from alcohol-insoluble residue (AIR) by boiling in 0.5% (w/v) ammonium oxalate buffer at 100°C. The content of galacturonic acid (GalA) was used to represent the pectin content and was determined colorimetrically by the metahydroxydiphenyl assay adopted from Blumenkrantz and Asboe-Hansen (1973) and from Louve et al. (2011) (for detailed protocol, please refer to Xu et al., 2011b). AIR was prepared according to the method described by Louve et al. (2011).

Classical AGPs were extracted according to Lamport and Varnai (2013) with minor modification. In brief, samples were blended in liquid nitrogen and stirred in 2% CaCl_2_ (w/v) at room temperature (RT) for 2–3 h. Supernatant was collected after centrifugation for 30 min at 10,000× g at RT. The AGP content in extract was estimated colorimetrically at 457 nm using 100 μl aliquots after precipitation with β-D-glucosyl-Yariv reagent (Biosupplies, Australia, 1 mg/ml in 2% CaCl_2_) and dissolving with 20 mM NaOH. The remaining supernatant was precipitated with a slight excess β-D-glucosyl-Yariv reagent, air dried and re-suspended in ddH_2_O.

### Immuno-fluorescence labeling and immuno-dot analysis of AGPs

Fixation and immuno-labeling were performed according to the method described by Xu et al. (2011a,b) with minor modifications. Briefly, the collected samples were fixed in 3.7% (v/v) formaldehyde in a buffer containing 1 mmol L^−1^ CaCl_2_ in 50 mmol L^−1^ piperazine-N,N'-bis(2-ethanesulfonic acid) (pH 7.4) (Eder and Lütz-Meindl, 2008) at RT for 1 h. Thin sections (8 μm) were mounted on microscope slides coated with 0.2% (w/v) polyethylenimine. All of the other immuno-labeling procedures were performed as described by Xu et al. (2011a).

Pectin prepared as described above was used for immuno-dot blots using JIM4, JIM8, CCRC-M32, and PN16.4B4 antibodies while AGPs were detected using JIM13, JIM14, JIM15, JIM16, MAC207, LM2, and LM14 antibodies. The concentration was adjusted to 1 mg/ml and 0.05 mg/ml for pectin (as 10 μl drops) and AGPs (as 5 μl drops) respectively before spotted onto nitrocellulose membrane by a micropipette. The membrane with dots was air dried at RT for 1 h. Assays with all antibodies were carried out as described by Willats et al. (2000). After the final wash, the membrane was developed in 3, 3'-diaminobenzidine tetrahydrochloride kit from TCI (Shanghai) Development Co., Ltd.

### Antibodies used

Primary antibodies used in this study and their antigens are listed in Table 1. LM antibodies were purchased from PlantProbes (UK) while all the other antibodies were from Complex Carbohydrate Research Center (Athens, USA). For immuno-fluorescence labeling, the secondary antibody for CCRC-M32, CCRC-M134 and PN16.4B4 primary antibodies was anti-mouse IgG-FITC (F9006, Sigma) while for the other antibodies was anti-rat IgG-FITC (F6258, Sigma). For immuno-dot blotting, anti-mouse IgG (whole molecule)-POD (A4416, Sigma) was used as a secondary antibody for CCRC series of primary antibodies as well as for PN16.4B4 antibody, and anti-rat IgG (whole molecule)-POD (A5795, Sigma) secondary antibody was used for all the others.

**Table 1:** **Antibodies used and their corresponding antigens**.

**Antibody**	**Antigen**	**References**
CCRC-M32	AG	Pattathil et al., 2010
CCRC-M134	AG	Pattathil et al., 2010
LM2	β-D-Gl*cp*A, AGP	Yates et al., 1996; Smallwood et al., 1996
LM14	AGP	Moller et al., 2008
MAC204	AGP	Bradley et al., 1988; VandenBosch et al., 1989; Pattathil et al., 2010
MAC207	β-D-Gl*cp*A-(1→3)-α-D-GalpA-(1→2)-L-Rha, AGP	Bradley et al., 1988; Yates et al., 1996
MAC265	Arabinogalactan protein-extensin	VandenBosch et al., 1989; Pattathil et al., 2010
MAC266	Arabinogalactan (AG)	Pattathil et al., 2010
PN16.4B4	Carbohydrate portion of AGP	Norman et al., 1986; Pattathil et al., 2010
JIM4	AGP	Yates et al., 1996
JIM8	AG	Pennell et al., 1991; McCabe et al., 1997
JIM13-16	AGPs	Knox et al., 1991; Yates et al., 1996
JIM17	AG/AGP	Yates et al., 1996
JIM101	AGP	Pattathil et al., 2010

### Quantification of fluorescence signal

For quantification of fluorescence signal, the integrated density (at least from three independent biological replicates) was measured with Image J 1.44 software. The fluorescence signal was selected using appropriate threshold levels for individual sections. Equal threshold levels were applied for each replicate, treatment and genotype for certain epitope to ensure exact comparison of cultivars and treatments.

### A histological examination of the banana leaf

The sections were prepared as described above. The histological examination was performed by staining the de-waxed sections with Coomassie brilliant blue G250 according to the method described by Ma et al. (2012).

### Statistical analysis

Statistical analyses were performed using ANOVA by statistics program SPSS 10.0 for Windows (SPSS Inc., Chicago, IL). At least three biological and two technical replicates were used in these experiments. Data are expressed as mean ± SD. Differences between cultivars for each LT treatment were statistically evaluated using Student's *t*-test.

## Results

### The chilling symptomes on banana leaves under LT stress

**Figure 1:**
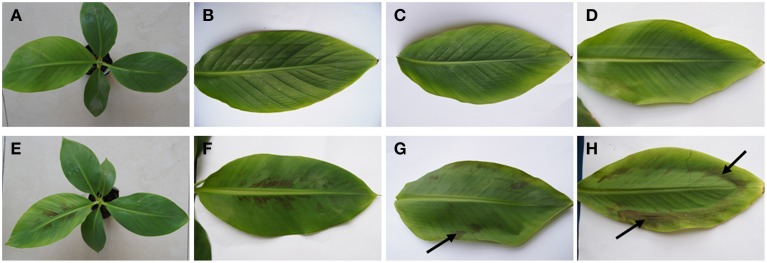
**Different responses of tolerant and sensitive banana (*Musa* spp.) genotypes to low temperature stress**. **(A,B)** The control plant and the youngest fully-developed leaf of *Musa* spp. ABB Dajiao (chilling-tolerant genotype). **(C)** The youngest fully-developed leaf of the tolerant genotype incubated at 10°C for 3 days. **(D)** The youngest fully-developed leaf of the tolerant genotype incubated at 7°C for 3 days, showing a slight loss in plant cell turgor. **(E,F)** The control plant and the youngest fully-developed leaf of *Musa* spp. AAA cv. Baxijiao (chilling-sensitive genotype). **(G)** The youngest fully-developed leaf of the sensitive genotype incubated at 10°C for 3 days, showing hydrophanous spots appearing on the leaf (arrows). **(H)** The youngest fully-developed leaf of the sensitive genotype incubated at 7°C for 3 days, showing more severe chilling injury symptom.

As shown in Figure 1, three-month-old banana seedlings of LT tolerant and sensitive cultivars showed different responses to the LT stress. Hydrophanous spots started to appear on the youngest fully-developed leaf of the sensitive genotype when the temperature dropped to 10°C. The symptoms of chilling injury became more severe and the leaf became partially wilt when the temperature decreased to 7°C. On the other hand, nearly no chilling injury symptom could be observed in the tolerant genotype, except very light loss of cell turgor at 7°C.

### A cross section of the banana leaf

**Figure 2:**
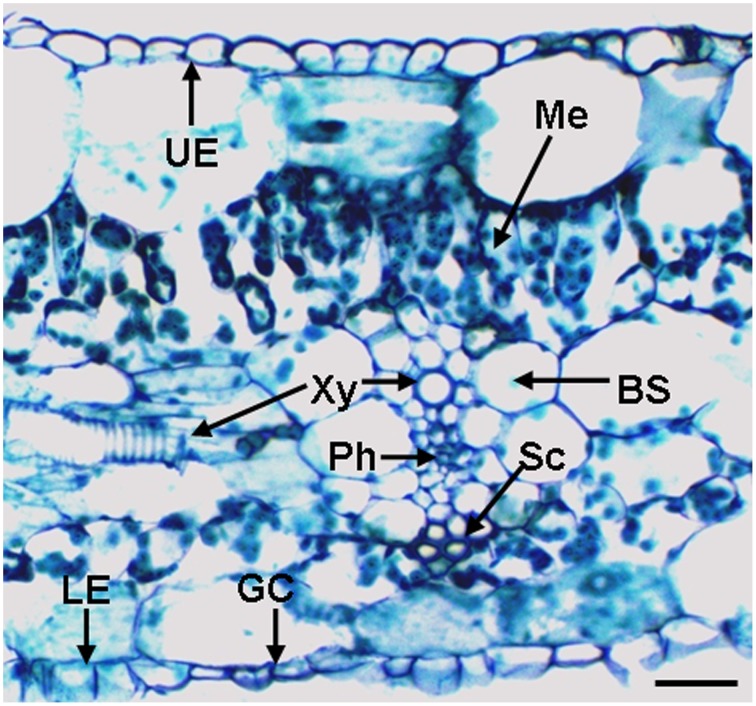
**A cross-section through the lateral vein of a banana (*Musa* spp.) leaf**. BS, bundle sheath; GC, guard cells; LE, lower epidermis; Me, mesophyll; Ph, phloem; Sc, sclerenchyma cells; UE, upper epidermis; and Xy, xylem vessel. The bar represents 50 μm.

A representative cross-section of a banana leaf is shown in Figure 2. This histological observation shows monolayers of upper epidermis and the lower epidermis, stomata located in the lower epidermis and a vascular bundle with xylem and phloem cells. The bundle sheath consists of a single layer of large cells enclosing the vein. Cells with a thick cell wall above or beneath the vein are sclerenchyma cells. This observation also shows mesophyll cells, including tightly packed cells with a cylindrical shape called the palisade layer and loosely packed, irregularly shaped spongy mesophyll (beneath the palisade layer). For representative cross-section of a banana root (*Musa* spp. AAA) in the elongation zone, please refer to Figure S1 of Ma et al. (2013).

### The subcellular distribution of AGPs in the cross section of the banana leaves

To reveal the spatial distribution of individual AGP components in banana leaves, a systematic immuno-fluorescence labeling by employing 17 monoclonal antibodies that recognize the epitopes of individual AGPs was carried out in the present study. Individual AGP components recognized by 11 out of these 17 antibodies showed different distribution patterns.

#### The JIM4, JIM14, JIM16, CCRC-M32, LM2, LM14, and MAC207 epitopes

**Figure 3:**
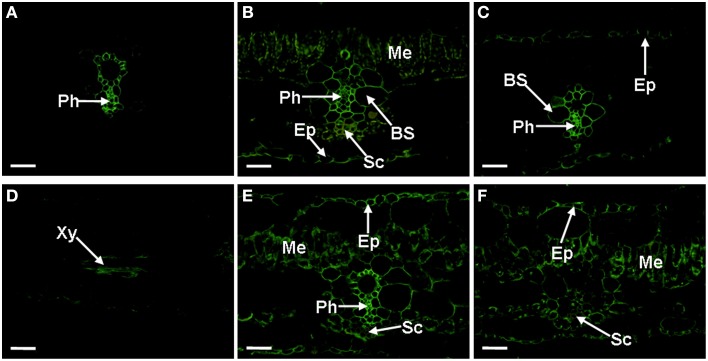
**Localization and distribution of AGPs recognized by JIM4, JIM14, JIM16, LM2, and LM14 antibodies in banana (*Musa* spp. AAA) leaves**. Cross-sections through the lateral vein are presented for all cases. **(A)** The JIM4 epitopes present in the phloem cells, showing a ring structure. **(B)** The JIM14 signal, mainly present in the vein, the sclerenchyma cells of the lower epidermis side, the phloem cells, and weak one also appeared in the mesophyll cells and the epidermal cells. **(C)** The JIM16 signal appeared in most cell types of the vascular bundle and the epidermal cells, showing a relatively stronger signal in the phloem. **(D)** Signal of JIM16 antibody, selected as the representative observed in the xylem cells between the veins for JIM4, JIM14, and JIM16 antibodies. **(E)** The LM2 signal, showing a strong signal appeared primarily in the vein (the phloem and the bundle sheath), a medium-strength signal in the mesophyll cells and the epidermal cells. **(F)** The LM14 signal, showing signal appeared primarily in the mesophyll cells, the epidermal cells and the phloem but not in the vein. AGPs, arabinogalactan proteins; BS, bundle sheath; Ep, Epidermis; Me, mesophyll; Ph, phloem; Sc, sclerenchyma cells; and Xy, xylem vessel. The bar represents 50 μm.

The JIM4 antibody binds β-GlcA-(1, 3)-α-GalA-(1, 2)-Rha, a plant cell wall AGP. In *Musa* spp. AAA Baxijiao leaves, this epitope is primarily localized in the phloem cells around the biggest xylem vessel of the vein, forming a ring structure (Figure 3A). JIM14 antibody signal primarily appeared in the vein (the phloem and the bundle sheath). The mesophyll cells and the epidermal cells were also weakly labeled (Figure 3B). The JIM16-binding AGP was located in nearly most cell types of the vascular bundle, including Kranz-type cells, the phloem and the bundle sheath cells, with a relatively stronger signal in the phloem. The epidermal cells (including the upper and lower epidermis) were also weakly labeled (Figure 3C). The labeling pattern of CCRC-M32 antibody was similar to that of JIM16 antibody, but the abundance was much lower (data not shown). All the three antibodies described above (JIM4, JIM14, and JIM16), also showed reactivity in the xylem cells between the veins (e.g., Figure 3D). The LM2 antibody binds to the β-D-Gl*cp*A of AGPs. A strong signal of this antibody appeared primarily in the vein (the phloem and the bundle sheath), while a signal of medium-strength appeared in the mesophyll and the epidermal cells. The sclerenchyma cells were not labeled (Figure 3E). Similarly, the LM14 (Figure 3F) and MAC207 (data not shown) binding AGP were also present in the mesophyll cells and the epidermal cells but not in the sclerenchyma cells. However, nearly no LM14 signal could be observed in the phloem, the ring structure (Figure 3F).

#### The JIM8, JIM13, JIM15, and PN16.4B4 epitopes

**Figure 4:**
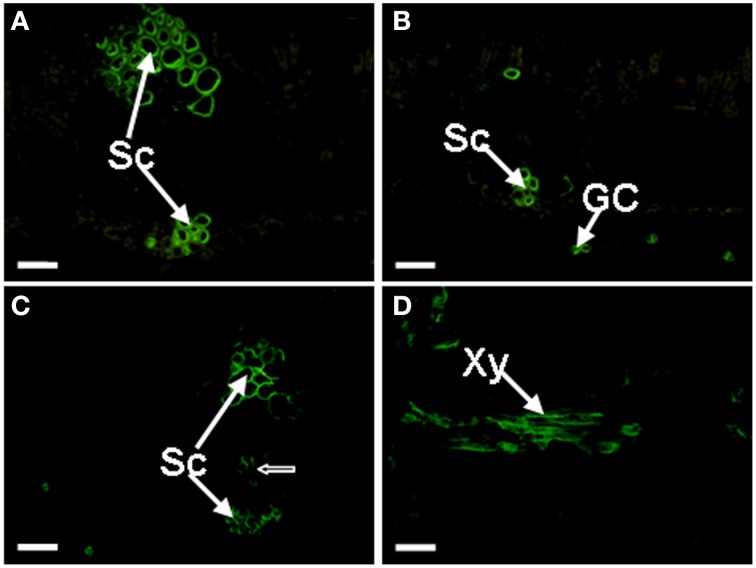
**Localization of JIM8 epitopes in banana (*Musa* spp.) leaves**. Cross-sections through the lateral vein are presented for all cases. **(A)** The epitopes appeared in the sclerenchyma cells of *Musa* spp. ABB Dajiao (chilling-tolerant genotype). **(B)** The epitopes appeared also in the GCs in both genotypes. **(C)** The epitopes appeared in *Musa* spp. AAA cv Baxijiao (chilling-sensitive genotype), exhibiting some cells of the vascular bundle (an arrow) were also labeled besides the sclerenchyma. **(D)** The epitopes observed in some xylem vessel in both genotypes. GC, guard cells; Sc, sclerenchyma cells; and Xy, xylem vessel. The bar represents 50 μm.

The JIM8 antibody binds a carbohydrate (galactose-rich) epitope of AGPs. JIM8 labeled epitopes were very abundant in the sclerenchyma cells of *Musa* spp. ABB Dajiao leaves (Figure 4A). In addition to the sclerenchyma cells, the signal could also be observed in the guard cells of the lower epidermis (Figure 4B). A difference was observed between chilling sensitive and tolerant cultivars. As shown in Figure 4C, some cells of the vascular bundle in chilling sensitive *Musa* spp. AAA Baxijiao were also labeled but this was not the case in chilling tolerant *Musa* spp. ABB Dajiao. Similar to JIM16 binding AGPs, the JIM8 antigen also appeared occasionally in the xylem cells between the veins (Figure 4D). The labeling patterns of JIM13 and JIM15 antibodies in the control banana leaves were similar to that of JIM8 in both genotypes and showed also preferentially sclerenchyma and guard cell localization and the same differences between genotypes. However, the labeling by JIM15 antibody was weaker and more bundle sheath cells of *Musa* spp. AAA Baxijiao were immunolabeled with JIM13 antibody when compared to JIM8 (data not shown). The PN16.4B4 antibody recognizes an epitope in the carbohydrate portion of AGP. The labeling pattern of this antibody in both genotypes was sclerenchyma and guard cell specific and resembled that of JIM8 in *Musa* spp. ABB Dajiao showing no signal in vascular bundles. However, the signal in guard cells was much less frequently observed (data not shown).

#### Other AGP epitopes

Negligible or very weak labeling was observed with 6 more antibodies that recognized different AGP components (CCRCM134, JIM17, JIM101, MAC204, MAC265 and MAC266) in the leaf sections of both banana genotypes (data not shown). This finding suggested that the above epitopes are under the immuno-labeling sensitivity threshold and are thus underrepresented in the banana leaves.

### Spatio-temporal changes in individual AGPs in banana leaves

#### The JIM4 and JIM16 epitopes

**Figure 5:**
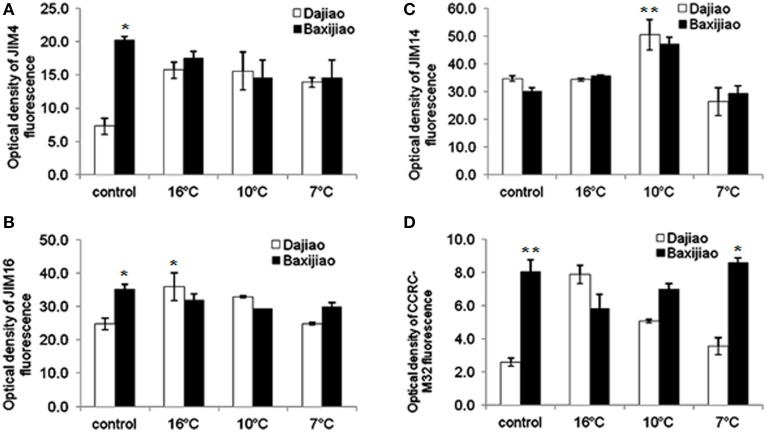
**The optical density of the fluorescence signal of arabinogalactan proteins labeled by JIM4, JIM16, JIM14, and CCRC-M32 antibodies in banana (*Musa* spp.) leaves after low temperature stress**. **(A)** JIM4; **(B)** JIM16; **(C)** JIM14; **(D)** CCRC-M32. Dajiao: a *Musa* spp. ABB cultivar, chilling-tolerant; Baxijiao: a *Musa* spp. AAA cultivar, chilling-sensitive. Values marked with star were considered significant at *P* < 0.05 while values marked with two stars were considered significant at *P* < 0.01.

As shown in Figure 5A, in the control plants, JIM4 antibody provided a substantially more intensive signal (nearly 3 fold) in chilling sensitive *Musa* spp. AAA Baxijiao than *Musa* spp. ABB Dajiao. However, the epitope abundance in *Musa* spp. ABB Dajiao increased by approximately 2.5 times when the temperature dropped to 16 and 10°C. When the temperature dropped further, the epitope abundance decreased a little but was still 1.8 fold higher than that of the control in this genotype. On the other hand, the JIM4 epitope abundance decreased slightly under these conditions in *Musa* spp. AAA Baxijiao, remaining at the same level as that in *Musa* spp. ABB Dajiao at 7°C. Changes in the abundance of JIM16 epitope in banana leaves under LT stress were similar to that of the JIM4 antibody (Figure 5B). These different changes in the antigen levels in two genotypes were mainly due to the intensity changes in the phloem cells (Figure S1).

#### The JIM14 and CCRC-M32 epitopes

The changes in the epitope levels of JIM14 antibody in LT treated banana leaves were similar in both genotypes. No obvious change was observed in the signal intensity when the temperature dropped to 16°C (Figure 5C, Figures S2A,B,E,F). At 10°C, the epitope in the mesophyll cells and sclerenchyma cells became more abundant (Figures S2C,G), resulting in 1.5 fold of increase in optical density compared to the control (Figure 5C). However, when the temperature dropped to 7°C, most of the signal disappeared and the epitope levels were even lower than the signals in the controls (Figure 5C, Figures S2D,H).

Though the labeling pattern of CCRC-M32 antibody was nearly the same was that of JIM16 antibody, the fluorescence signal of this antigen [(1→6)-β-D-galactan I] appeared to be much weaker and it was hardly detectable in *Musa* spp. ABB Dajiao (chilling-sensitive) (Figure 5D). The epitope levels of this antibody were only 10.7% and 22.7% of JIM16 antibody in *Musa* spp. ABB Dajiao and *Musa* spp. AAA Baxijiao, respectively (Figure 5D). When the temperature dropped from 25 to 16°C, the abundance of this epitope increased more than 3 fold in the chilling-tolerant genotype and decreased by 1.37 fold in the chilling-sensitive genotype (Figures 5B,D). After stronger LT stress, the epitope levels gradually returned to the control levels for both genotypes (Figure 5D).

#### The LM2 and LM14 epitopes

**Figure 6:**
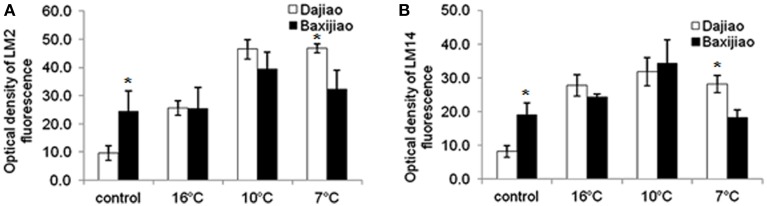
**The optical density of the fluorescence signal of arabinogalactan proteins labeled by LM2 and LM14 antibodies in banana (*Musa* spp.) leaves after low temperature stress**. **(A)** LM2: **(B)** LM14. Dajiao: a *Musa* spp. ABB cultivar, chilling-tolerant; Baxijiao: a *Musa* spp. AAA cultivar, chilling-sensitive. Values marked with star were considered significant at *P* < 0.05 while values marked with two stars were considered significant at *P* < 0.01.

The signal for LM2 antibody was relatively weak in the control chilling tolerant *Musa* spp. ABB Dajiao (Figure 6A, Figure S3A). The epitope abundance increased gradually in response to LT, reaching maximum at 7°C and showing 4.8 fold increase compared to the control. It was newly induced by LT in the mesophyll cells (Figures S3B–D). The signal in control of *Musa* spp. AAA Baxijiao was nearly 3 times stronger than that of *Musa* spp. ABB Dajiao (Figure 6A). The epitope abundance in *Musa* spp. AAA Baxijiao did not change when the temperature dropped from 25 to 16°C, however 10°C caused 1.6 fold increase in its abundance, and peaked at 10°C (Figure 6A, Figures S3E–H). As a result, the epitope abundance in the tolerant genotype at 10 and 7°C was higher than in the sensitive genotype, although the opposite trend was observed in control plants (Figure 6A, Figure S3).

Similar to LM2 antibody, the signal of LM14 in *Musa* spp. ABB Dajiao control was also very weak. The antigen abundance in this tolerant genotype was only 43% as high as in *Musa* spp. AAA Baxijiao, the sensitive genotype (Figure 6B, Figure S4A). LT treatment resulted in a dramatic increase (3 fold after 16°C, 4 fold after 10°C, 3 fold after 7°C) in the epitope abundance in *Musa* spp. ABB Dajiao, and new signals also appeared in the veins (the phloem and bundle sheath cells) (Figure 6B, Figures S4A–D). The epitope also accumulated when the temperature dropped to 16 and 10°C in *Musa* spp. AAA Baxijiao (Figure 6B, Figures S4E–G). However, the epitope abundance decreased to the level of the control when the temperature dropped to 7°C (Figure 6B, Figure S4H).

#### The JIM8, JIM15, and PN16.4B4 epitopes

**Figure 7:**
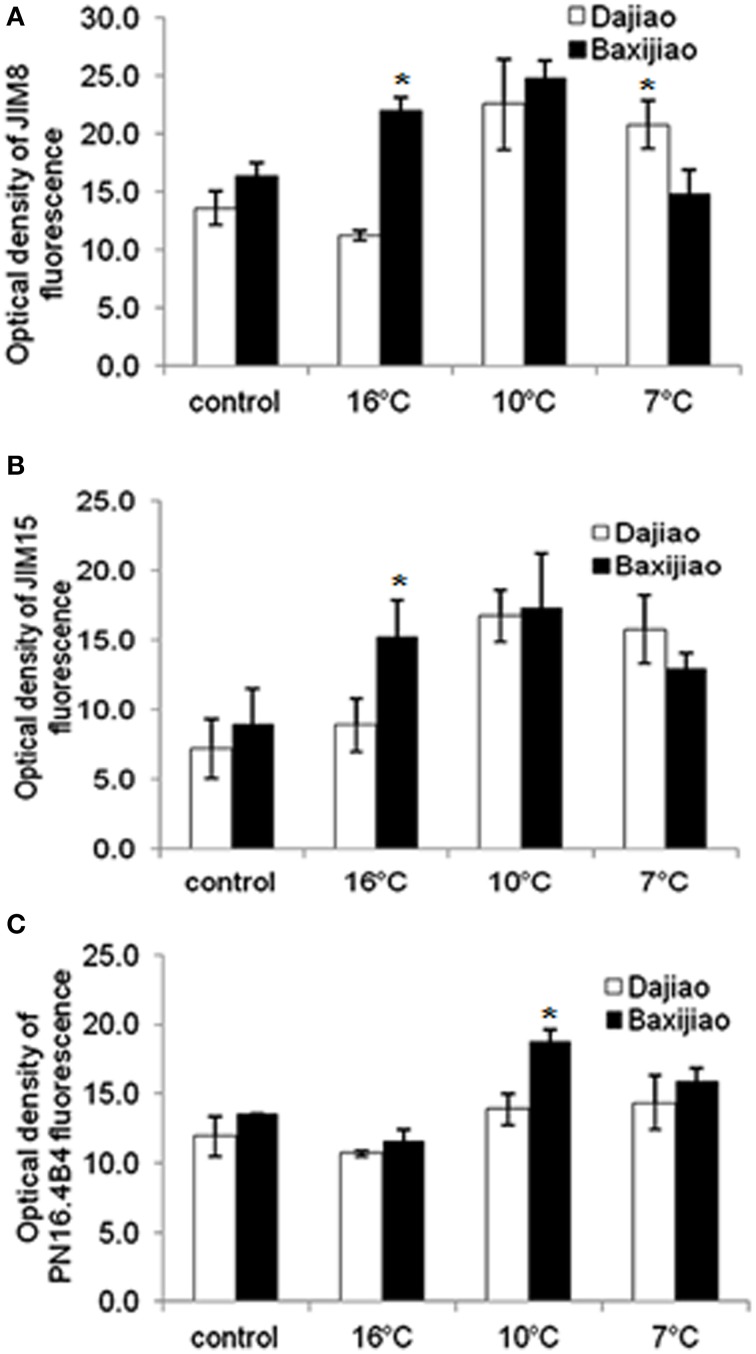
**The optical density of the fluorescence signal of arabinogalactan proteins labeled by JIM8, JIM15 and PN16.4B4 antibodies in banana (*Musa* spp.) leaves after low temperature stress**. **(A)** JIM8; **(B)** JIM15; **(C)** PN16.4B4. Dajiao: a *Musa* spp. ABB cultivar, chilling-tolerant; Baxijiao: a *Musa* spp. AAA cultivar, chilling-sensitive. Values marked with star were considered significant at *P* < 0.05 while values marked with two stars were considered significant at *P* < 0.01.

Figure 7A showed that the abundance of JIM8 epitopes in the sensitive genotype was slightly (18.5%) higher than in the tolerant genotype in the control plants. The response of sensitive genotype *Musa* spp. AAA Baxijiao to LT differed from *Musa* spp. ABB Dajiao, the tolerant genotype. While a slight decrease (17%) was observed in the epitope level of chilling-tolerant genotype when the temperature decreased from 25 to 16°C, a clear increase (1.6 fold) in the signal intensity appeared when the temperature dropped further. The signal in *Musa* spp. AAA Baxijiao increased when the temperature dropped from 25 to 16°C, and this increase also occurred at 10°C (Figure 7A). However, the epitope level decreased to the level of the control again when the temperature dropped down to 7°C (Figure 7A). Finally, the abundance of this epitope in the sensitive genotype was lower than in the tolerant genotype at 7°C which was opposite to that at 25°C.

Similar to JIM8, the labeling by JIM15 antibody was also weaker in the control plants of the tolerant genotype than in the sensitive one (Figure 7B). However, the change in epitope abundance was nearly the same in both tolerant and sensitive genotypes. The LT treatment resulted in a signal strength increase in banana leaves and peaked at 10°C. The optical density increased 1.65 and 1.51 fold in the tolerant and sensitive genotype, respectively (Figure 7B). When compared with the sensitive genotype, the tolerant genotype started to greatly accumulate the antigen under stronger LT stress. Notably, the signal in the tolerant genotype was stronger at 7°C when compared with the sensitive genotype, with the opposite signal trend being observed at higher temperatures (25, 16, and 10°C) (Figure 7B).

In both genotypes, the intensity of PN16.4B4 epitope decreased slightly at 16°C followed by a gradual increase when the temperature further dropped (Figure 7C). As a result, the epitope level increased by decline of temperature in both genotypes (Figure 7C).

Abundance and localization changes of AGPs recognized by JIM16, JIM14, LM2 and LM14 antibodies, respectively in different cell types of LT treated banana leaves are provided in the Supporting Information Figures (Supplemental Figures S1–S4).

**Figure 8:**
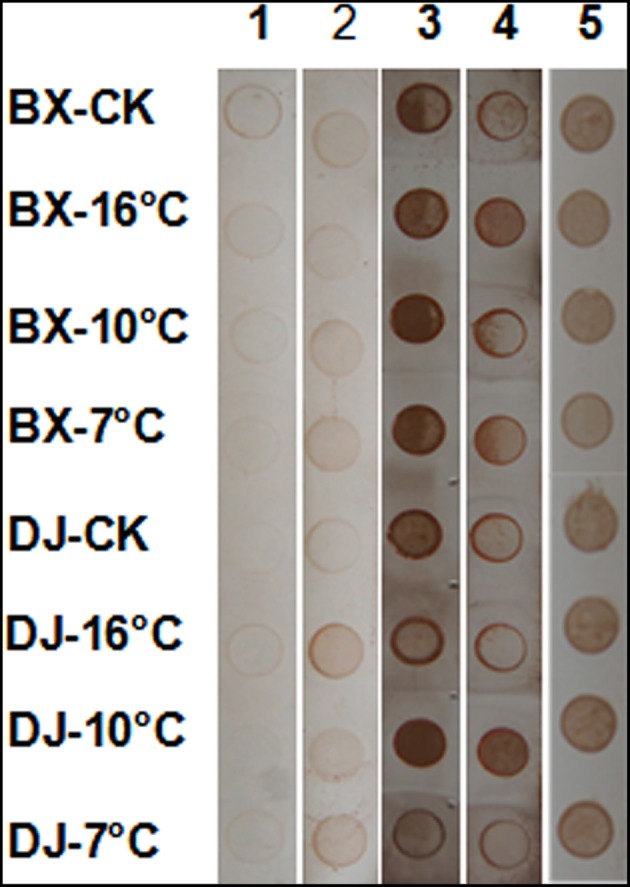
**Changes in the levels of individual AGPs in banana leaves exposed to different temperatures using pectin (lanes 1, 2) and AGP extracts (lanes 3–5) probed by immuno-dot analysis**. 1, CCRC-M32 antibody; 2, JIM4 antibody; 3, LM2 antibody; 4, LM14 antibody; 6, JIM16 antibody. BX, Baxijiao (a *Musa* spp. AAA cultivar, chilling-sensitive); DJ, Dajiao (a *Musa* spp. ABB cultivar, chilling-tolerant). Note much stronger labeling with AGP-specific antibodies LM2, LM14, and JIM16.

Immuno-dot analyses on leaf pectin and AGP extracts were also employed to confirm the results from immuno-fluorescence labeling. Figure 8 shows the abundance of pectins and AGPs immuno-reacting with CCRC-M32, JIM4, JIM16, LM2, and LM14 antibodies challenged with low temperature stress. Basically, the changes revealed by immuno-dot blotting were consistent with those from immuno-fluorescence labeling, especially for JIM4 and JIM16 antibodies.

### Changes in classical AGP levels in banana leaves

Leaves of two banana genotypes showed distinct changes in the level of classical AGPs under LT stress (Table 2). In the control leaves, the AGP content of chilling tolerant *Musa* spp. ABB cv Dajiao was significantly higher than in the chilling sensitive *Musa* spp. AAA cv Baxijiao. When the temperature dropped from 25 to 16 and 10°C, both genotypes accumulated AGPs. However, when the temperature further dropped to 7°C, the AGP level in *Musa* spp. AAA cv Baxijiao decreased to the level of the control which was not the case for the chilling-tolerant genotype, *Musa* spp. ABB cv Dajiao.

**Table 2:** **Changes in classical AGPs (μg/g. fw) in banana (*Musa* spp.) after low temperature stress**.

**Genotype/temperature**	**Control**	**16°C**	**10°C**	**7°C**
*Musa* spp. AAA cv. Baxijiao	28.4 c	36.8 b	45.6 a	30.4 c
*Musa* spp. ABB cv. Dajiao	37.4 b	43.0 a	43.2 a	45.8 a

*Values represent averages from three biological replicates. Different letters after values mean that they are significantly different using Duncan's multiple range test at P < 0.05 after angular transformation of the data*.

### The sub-cellular distribution of AGPs in cross sections of banana roots

**Figure 9:**
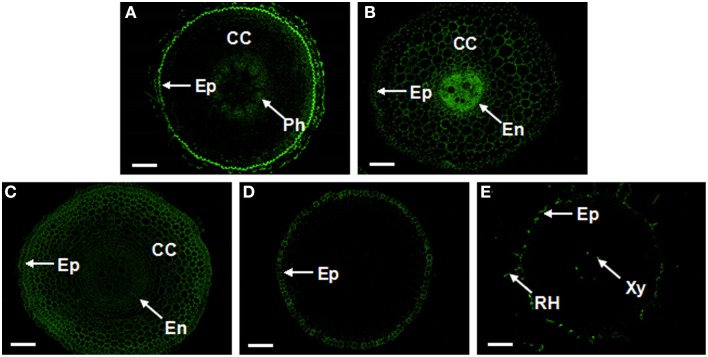
**Localization and distribution of AGPs recognized by JIM16, LM2, LM14, and JIM13 antibodies in cross-sections from banana (*Musa* spp. AAA) roots**. **(A)** The JIM16 epitopes present in the outer part of epidermis, cortical cells adjacent to the epidermis and in phloem cells; **(B)** The strongest signal of LM2 in the central cylinder enclosed by the endodermis, followed by cortical cells and the epidermis; **(C)** The LM14 signal appeared in most cell types, showing a relatively strong signal in the epidermis and cortical cells; **(D,E)** The JIM13 signal in the epidermis of the meristematic part of the root **(D)** and in root hairs and xylem cells in matured root zone **(E)**. AGPs, arabinogalactan proteins; CC, cortex cells; En, endodermis; Ep, Epidermis; Ph, phloem; RH, root hair; and Xy, xylem vessel. The bar represents 100 μm.

Abundances of AGPs in banana roots differed from leaves. CCRC-M32, JIM4, JIM8, JIM14 and JIM15 antibodies provided only weak labeling in the roots. Distributions of individual AGPs in the cross-sections of banana roots are provided in Figure 9. The strongest signal was observed using JIM16 antibody in the outer part of epidermis, slightly weaker JIM16 labeling was observed in 2–3 layers of cortical cells (adjacent to the epidermis) and by phloem cells (Figure 9A). The AGP epitope recognized by LM2 antibody was distributed nearly in all cell types, with very high level in the central cylinder and relatively lower level in the endodermis (Figure 9B). The AGP epitope immuno-reacting with LM14 antibody also appeared in all types of cells in the root cross-section, while intensity of the labeling gradually decreased from the epidermis to the central cylinder (Figure 9C). Although weaker, the labeling pattern with MAC207 antibody was the same as in the case of LM14 antibody (data not shown). AGPs recognized by JIM13 antibody mainly appeared in the epidermis within the meristematic zone of banana root (Figure 9D). In the differentiation root zone, they can be found also in root hairs and xylem cells (Figure 9E). The immunolabeling with PN16.4B4 antibody was visible in the root hairs and the epidermal cells in the differentiation root zone, similarly to the JIM13 antibody. But no obvious signal of PN16.4B4 antibody could be observed in the meristematic zone (data not shown).

### Dynamics of individual AGPs in response to LT in banana roots

**Figure 10:**
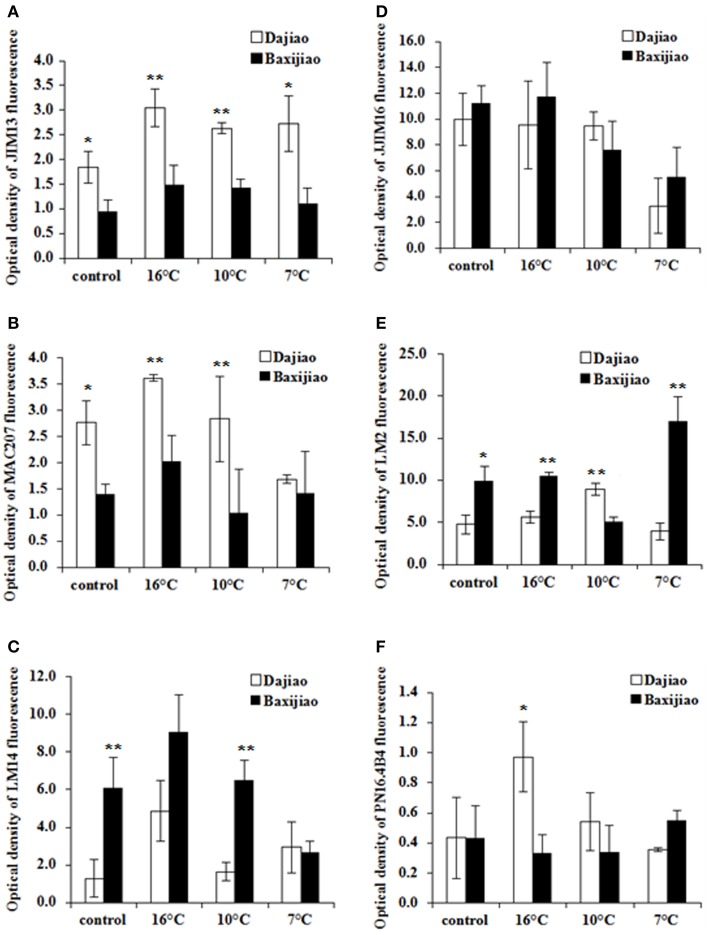
**The optical density of the fluorescence signal of arabinogalactan proteins labeled by JIM13, MAC207, LM14, JIM16, LM2, and PN16.4B4 antibodies in banana (*Musa* spp.) roots after low temperature stress**. **(A)** JIM13; **(B)** MAC207; **(C)** LM14; **(D)** JIM16; **(E)** LM2; **(F)** PN16.4B4. Dajiao: a *Musa* spp. ABB cultivar, chilling-tolerant; Baxijiao: a *Musa* spp. AAA cultivar, chilling-sensitive. Values marked with star were considered significant at *P* < 0.05 while values marked with two stars were considered significant at *P* < 0.01.

Figure 10 shows changes in the levels of different types of AGP antigens in banana roots after LT treatment. In the control plants, the levels of antigen recognized by JIM13 and MAC207 antibodies were much higher in the tolerant genotype when compared to the sensitive one (Figures 10A,B). Both genotypes accumulated these two AGP epitopes mainly after mild LT treatment (16°C) while they decreased at lower temperatures (10°C and 7°C). LM14 showed very similar dynamics in response to LT as the previous antigens. Unlike to JIM13 and MAC207, the levels of antigen recognized by LM14 antibody were significantly lower in control or 10°C-exposed plants of the tolerant genotype compared to the sensitive genotype (Figure 10C). On the contrary, no obvious changes in the level of JIM16 epitope were observed between two genotypes regardless of LT stress, as both showed decrease in signal intensity at 7°C (Figure 10D). Levels of LM2 epitope showed the highest variability. They were lower in the tolerant cultivar under control conditions, at 16°C and at 7°C when compared to the sensitive cultivar (Figure 10E). At 10°C, however, the same epitope was more abundant in the tolerant cultivar. Finally, the level of the epitope recognized by PN16.4B4 antibody increased at 16°C in the tolerant genotype (Figure 10F).

## Discussion

### The extracellular distribution of AGPs in banana leaves and roots

As mentioned above, immuno-histochemical technique has been widely used to study the distribution and function of plant cell wall proteins in plant growth and development. These studies were performed on roots (Smallwood et al., 1994; Šamaj et al., 1998, 1999b), embryogenic cells or embryos (Šamaj et al., 1999a, 2008; Pan et al., 2011; Xu et al., 2011a), shoot apical meristems (Zhang et al., 2008), and flower components (Chen et al., 2008; Losada and Herrero, 2014). However, information about leaves is very limited in this regard. In the present study, we found that most of the AGP components of the plant cell walls were located in the vein (the epitopes of CCRC-M32, JIM4, JIM14, and JIM16 antibodies) and sclerenchyma cells (the epitopes of JIM8, JIM13, JIM15, and PN16.4B4 antibodies) of banana leaves. On the other hand, MAC207, LM2, and LM14 antibodies strongly labeled mesophyll cells.

In banana roots, AGP epitopes of MAC207, LM2, and LM14 antibodies appeared in high abundance in the epidermis, cortical cells and central cylinder. The JIM16 antigen mainly localized in the epidermis and phloem cells. AGPs recognized by JIM13 and PN16.4B4 antibodies mainly appeared in the epidermis, the root hairs and xylem cells. AGPs showed also different abundance in banana roots as compared to leaves. For example, highly abundant AGP epitopes in leaves recognized by CCRC-M32, JIM4, JIM8, JIM14, and JIM15 antibodies gave weak signal in roots.

### The involvement of AGPs in banana tolerance against chilling stress

LT is one of the most important factors limiting the productivity and distribution of bananas in China (Huang et al., 1982; Wang and Liang, 1994; Chen et al., 1999; Xu et al., 2000; Yang et al., 2012). The appropriate and timely modification of the cell wall components, such as HRGPs, can help a plant to survive under rapidly fluctuating environmental conditions. In the present study, we not only determined the total classical AGPs in banana leaves, but also monitored the changes in the extracellular distribution and relative abundance of individual AGP groups by immuno-histochemical staining with 17 monoclonal antibodies that specifically recognize the different epitopes of individual AGPs. Immuno-dot analysis was applied to further confirm the results. The immuno-fluorescence labeling results clearly revealed that several types of plant cell wall AGPs were differentially altered under LT stresses as follows.

First, the accumulation of some AGP components was a common phenomenon in banana upon mild LT treatment (at 16 and/or 10°C). As shown in Table 2, both chilling sensitive and tolerant genotypes accumulated classical AGPs under LT treatment. Furthermore, most AGP epitopes which were detectable in the present study (antigens of the JIM8, JIM14, JIM15, LM2 and LM14 antibodies in banana leaves, antigens of JIM13, LM2, LM14, MAC207, and PN16.4B4 antibodies in banana roots) increased in both chilling-sensitive and tolerant banana genotypes under chilling stress. Similarly, Gong et al. (2012) found that the expression of *GhAGP31* (a cotton non-classical AGP) in cotton roots was markedly up-regulated by cold stress. Furthermore, the expression of the *GUS* gene as driven by the *GhAGP31* promoter was also dramatically enhanced in the roots of transgenic *Arabidopsis* seedlings under cold treatment. Additionally, the overexpression of *GhAGP31* in yeast and *Arabidopsis* significantly improved the freezing tolerance of yeast cells and the cold tolerance of *Arabidopsis* seedlings. During the cold hardening of winter wheat (*Triticum aestivum* L., cv. Mironovskaya 808), a transient appearance of 37-and 69-kD lectins, which were identified as AGPs, might indicate their involvement in triggering plant cell defense responses to LT stresses (Garaeva et al., 2006). The overexpression of flower specific PRP *OsPRP3* conferred the cold tolerance in rice (Gothandam et al., 2010).

Another important outcome of our study is that different types of plant cell wall AGPs were differentially modified in banana tissues under LT stress. In general, AGP epitopes showed four different responses to LT in leaves:

1. LM2, LM14, JIM8, and JIM15 AGP epitopes showed positive correlation of their abundance with the tolerance of the cultivars at lower temperatures. It indicates that they are important for banana tolerance to strong chilling stress.
2. The abundance of JIM16 and CCRC-M32 epitopes was higher in the tolerant cultivar at mild LT stress suggesting that these epitopes determines the tolerance of banana to milder LT stress.
3. The epitopes recognized by JIM14 and PN16.4B4 antibodies increased in both cultivars at 10 and/or 7°C, indicating that these two AGP components are chilling-responsive, but they likely do not contribute to banana tolerance to chilling injury.
4. In the control plants, the AGPs recognized by JIM8, JIM15, LM2, and LM14 antibodies exhibited much lower abundance in the chilling tolerant genotype when compared with the chilling-sensitive one; however, the abundances of these epitopes were reversed at 7°C. These results indicate that the tolerant genotype accumulates these AGPs faster under chilling stress. This finding is consistent with the earlier accumulation of soluble proteins and free proline in the leaves of the tolerant banana genotype under LT stress when compared with the sensitive genotype as found by our previous study (Chen et al., 1999).

The differential expression of different HRGP types under LT stress was also reported in rice, and the transcript levels of three AGP genes (*OsAGP3*, *OsAGP20*, and *OsAGP24*) were found to be up-regulated by all three stresses (cold, draft, and salt). However, another two fasciclin-like AGP genes (*OsFLA1* and *OsFLA4*) were down-regulated by cold stress (Ma and Zhao, 2010).

Third, the present study not only revealed the differential changes in AGP abundance, but also in the spatial distribution of AGPs in different cell types in LT treated banana leaves. For example, the accumulation of AGPs recognized by JIM4 and JIM16 was only observed in the phloem cells of the tolerant genotype (Figure 5, Figure S1). LT induced much more AGP epitopes of LM2 and LM14 antibodies in the tolerant genotype with comparison with the sensitive one, especially in the mesophyll cells (Figure 6, Figures S3, S4). Consistently, the symptom of chilling injury could be observed in the sensitive genotype at 10°C and 7°C but not in the tolerant one (Figure 1). The water-holding capacity of AGPs was suggested in the past (Clarke et al., 1979). Because LT limits water uptake by plants, this function of AGPs possibly could help bananas to maintain their cell turgor under LT and reduce the wilt damage caused by LT. The accumulation of AGPs in the phloem cells and mesophyll cells of LT treated banana leaves also could actively protect banana against chilling injury through reinforcing mechanical strength and integrity of cell walls.

In addition, the same AGP epitopes differed in their response to LT between banana leaves and roots. Furthermore, though both leaves and roots accumulated AGPs under LT treatment, the highest AGP levels were detected in roots at 16°C while in leaves it was at 10°C. This might indicate that banana roots started to accumulate AGPs earlier than leaves or eventually, that they were more sensitive to decreased temperatures. It has to be noted that differences between roots and leaves in AGP abundance and dynamics during LT stress could be also related to different developmental stages of the analyzed plant material.

### The cross –reactivities of antibodies binding AGPs

AGP glycan-specific antibodies have been broadly used to investigate AGPs in tissue culture and *in planta* (Seifert and Roberts, 2007; Ellis et al., 2010). Recently, it was assumed that AGPs could act as covalent cross-linkers in polysaccharide networks (Pattathil et al., 2010; Hijazi et al., 2014). Thus, epitopes recognized by some antibodies against AGPs used in the present study may not stand only for AGPs themselves. For example, JIM14 antibody in AG-2 clade binds to linear and branched arabinans and RG-I preparations from diverse plants but do not bind to larch arabinogalactan. The antibodies in AG-3 clade, such as JIM4, JIM8, and JIM15, not only strongly bind to gum Ghatti and gum Arabic, but also to pectic polysaccharide preparations from tomato and sycamore maple (Pattathil et al., 2010). Conclusively, the complex properties of AGPs make purification of individual AGPs difficult (Hijazi et al., 2014) and impossible to assign function to a single AGP (Tan et al., 2012). Fortunately, modern genetic and molecular techniques, such as genomic analyses, use of AGPs single or double mutants, RNAi and over-expressing lines, could offer the potential to unlock the mysteries of AGP structure and functions.

## Author contributions

CX planned experiments, YY, YW, EX, LX, XY, ZS, and CX performed experiments, CX, TT, JS, XL, and HC analyzed data and prepared data presentation, CX, TT, and JS wrote the manuscript. All authors read and approved the final manuscript.

## Conflict of interest statement

The authors declare that the research was conducted in the absence of any commercial or financial relationships that could be construed as a potential conflict of interest.

## Abbreviations

AGPs: arabinogalactan proteins
GalA: galacturonic acid
HRGPs: hydroxyproline-rich glycoproteins
LT: low temperature
PRPs: proline-rich proteins
RT: room temperature.
