# Supplementary material for: Variable content and distribution of arabinogalactan proteins in banana (Musa spp.) under low temperature stress
Source: Front Plant Sci. 2015 May 27;6:353. doi: 10.3389/fpls.2015.00353 (PMC4444754; doi:10.3389/fpls.2015.00353)
Supplement: Supplementary file 1 [file DataSheet1.PDF]

Supporting Information Figs S1–S4

Variable content and distribution of arabinogalactan proteins in banana (*Musa* spp.) under low temperature stress

Yonglian Yan[1], Tomáš Takáč[2], Xiaoquan Li[3], Houbin Chen[1], Yingying Wang[1], Enfeng Xu[1], Lin Xie[1], Zhaohua Su[1], Jozef Šamaj[2], Chunxiang Xu[1*]

Author for correspondence: Chunxiang Xu

Email: chxxu@scau.edu.cn

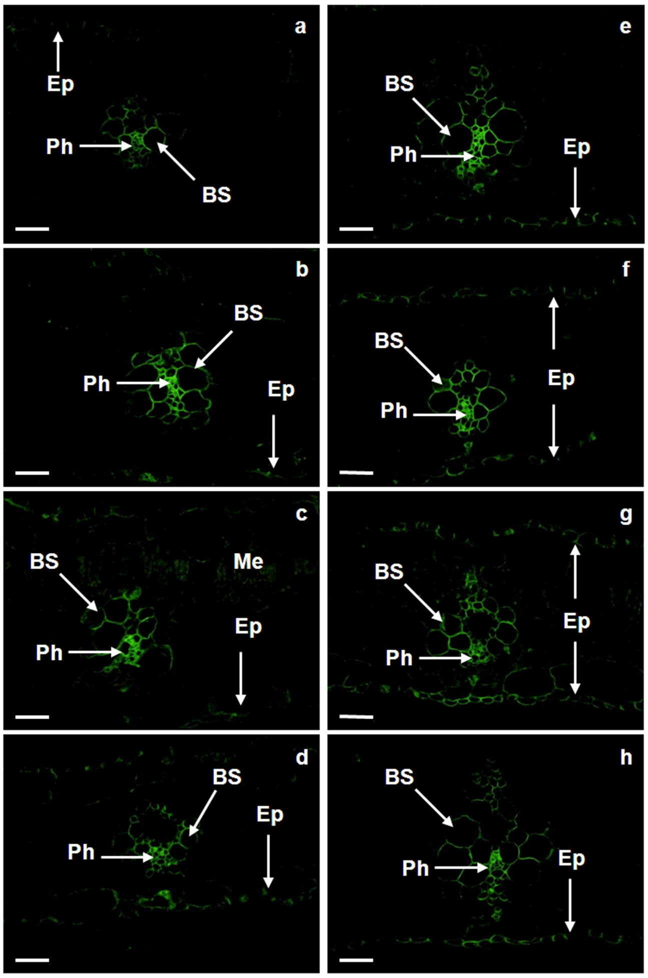

Fig. S1 Immunolocalization of the JIM16 epitope in banana (*Musa* spp.) leaves. In all cases, cross-sections through the lateral vein are presented. **(a, e)** The control plants exhibiting a much weaker signal in *Musa* spp. ABB Dajiao (a) than in *Musa* spp. AAA cv Baxijiao (e); **(b, f), (c, g)** and **(d, h)** Three days after incubation at 16, 10, and 7°C respectively. **(a-d)** *Musa* spp. ABB Dajiao (chilling-tolerant genotype) exhibiting increased epitope levels at 16 and 10°C but decreased to the level of the control at 7°C (especially in the phloem cells); **(e-h)** *Musa* spp. AAA cv Baxijiao (chilling-sensitive genotype) showing a slightly decreased epitope level at 16°C followed by a returned to that of the control when the temperature further dropped. BS bundle sheath, Ep epidermis, Me mesophyll, and Ph phloem. Bars represent 50 μm

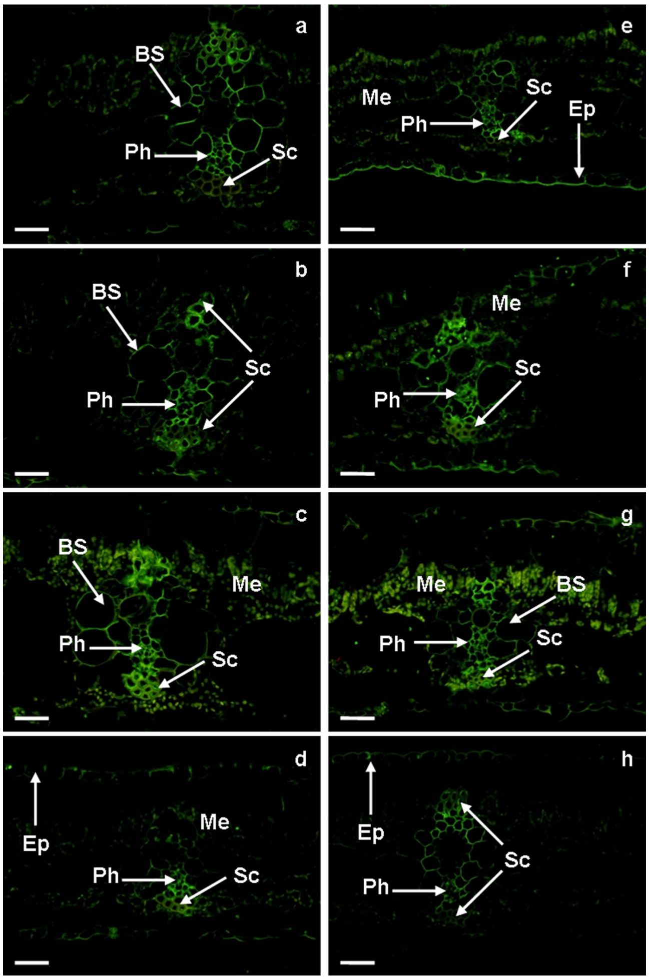

Fig. S2 Immunolocalization of the JIM14 epitope in banana (*Musa* spp.) leaves. Cross-sections through the lateral vein are presented in all cases. **(a-d)** *Musa* spp. ABB cv. Dajiao, **(e-h)** *Musa* spp. AAA cv. Baxijiao. **(a, e)** The control plants, exhibiting a medium-strength signal primarily distributed in the vein; **(b, f), (c, g)** and **(d, h)** Three days after incubation at 16, 10, and 7°C with an increased epitope level at 10°C in both genotypes. BS bundle sheath, Ep epidermis, Me mesophyll, Ph phloem, and Sc sclerenchyma. Bars represent 50 μm

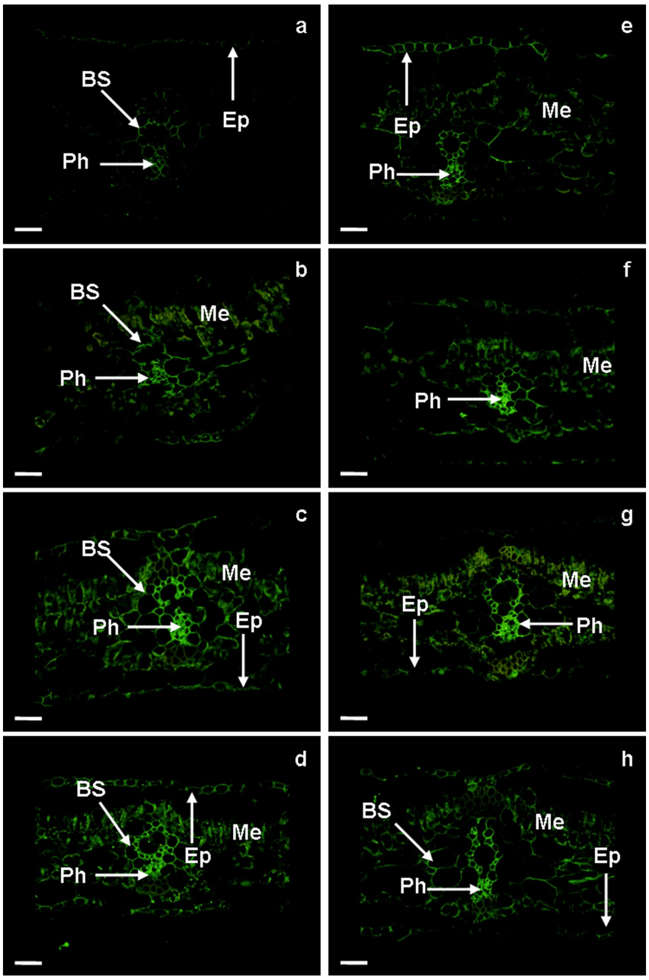

Fig. S3 Immunolocalization of the LM2 epitope in banana (*Musa* spp.) leaves. In all cases, cross-sections through the lateral vein are presented. **(a, e)** The control plants, exhibiting a stronger signal in the vein, the mesophyll and epidermis in *Musa* spp. AAA (chilling sensitive) (e) than in ABB (tolerant genotype) (a); (**b, f**), **(c, g)** and **(d, h)** Three days after incubating at 16, 10, and 7°C, there were increased epitope levels in the tolerant genotype from 16 to 7°C (b-d) while the epitope level started to increase at 10°C followed by a significant decrease at 7°C in the sensitive genotype (f-h). BS bundle sheath, Ep epidermis, Me mesophyll, and Ph phloem. Bars represent 50 μm

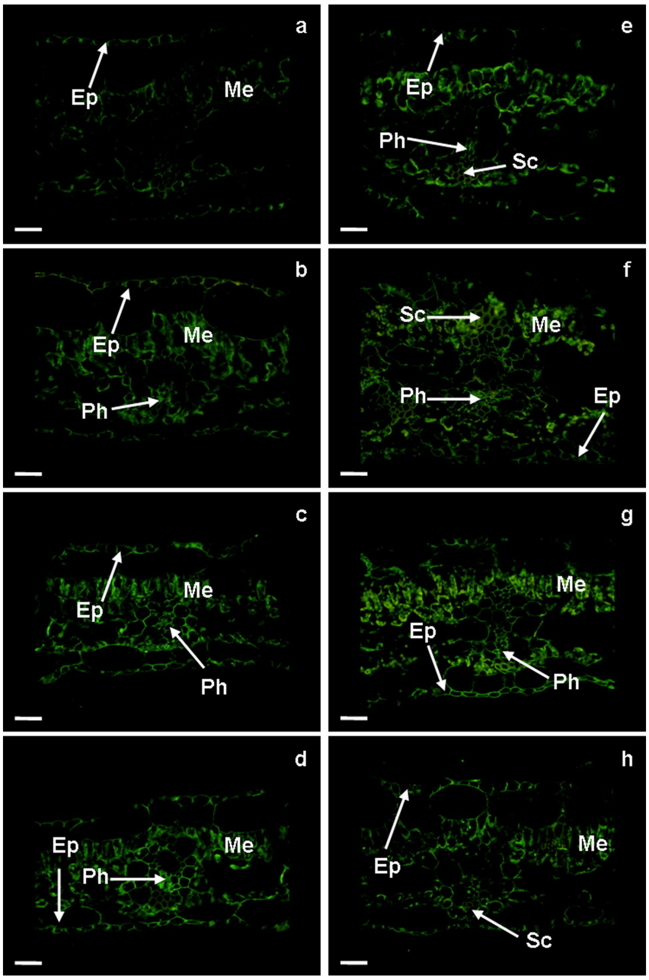

Fig. S4 Immunolocalization of the LM14 epitope in banana (*Musa* spp.) leaves. In all cases, cross-sections through the lateral vein are presented. **(a-d)** *Musa* spp. ABB cv. Dajiao, **(e-h)** *Musa* spp. AAA cv. Baxijiao. **(a, e)** The control plants with a stronger signal in the mesophyll and epidermis of *Musa* spp. AAA cv. Dajiao (chilling-sensitive) (e) than in ABB (tolerant) (a); **(b, f), (c, g)** and **(d, h)** Three days after incubation at 16, 10, and 7°C respectively, with increased epitope levels in both genotypes after LT treatment (b-d, f-g) except the level decreased to that of the control at 7°C only in the sensitive type (h). Ep epidermis, Me mesophyll, Ph phloem, and Sc sclerenchyma. Bars represent 50 µm
